# Comparing methods for the modelling of boundary-driven streaming in acoustofluidic devices

**DOI:** 10.1007/s10404-017-1865-z

**Published:** 2017-02-07

**Authors:** Junjun Lei, Peter Glynne-Jones, Martyn Hill

**Affiliations:** Faculty of Engineering and the Environment, University of Southampton, University Road, Southampton, SO17 1BJ UK

**Keywords:** Acoustic streaming, Boundary-driven streaming, Reynolds stress method, Limiting velocity method, Acoustofluidics, Acoustic boundary layer

## Abstract

Numerical simulations of acoustic streaming flows can be used not only to explain the complex phenomena observed in acoustofluidic manipulation devices, but also to predict and optimise their performances. In this paper, two numerical methods based on perturbation theory are compared in order to demonstrate their viability and applicability for modelling boundary-driven streaming flows in acoustofluidic systems. It was found that the Reynolds stress method, which predicts the streaming fields from their driving terms, can effectively resolve both the inner and outer streaming fields and can be used to demonstrate the driving mechanisms of a broad range of boundary-driven streaming flows. However, computational efficiency typically limits its useful application to two-dimensional models. We highlight the close relationship between the classical boundary-driven streaming vortices and the rotationality of the Reynolds stress force field. The limiting velocity method, which ignores the acoustic boundary layer and solves the outer streaming fields by applying the ‘limiting velocities’ as boundary conditions, is more computationally efficient and can be used for predicting three-dimensional outer streaming fields and provide insight into their origins, provided that the radius of curvature of the channel surfaces is much greater than the acoustic boundary layer thickness ($\delta_{v}$). We also show that for the limiting velocity method to be valid the channel scales must exceed a value of approximately 100 $\delta_{v}$ (for an error of ~5% on the streaming velocity magnitudes) for the case presented in this paper. Comparisons of these two numerical methods can provide effective guidance for researchers in the field of acoustofluidics on choosing appropriate methods to predict boundary-driven streaming fields in the design of acoustofluidic particle manipulation devices.

## Introduction

Acoustic streaming comprises steady, time-averaged fluid flows driven by acoustic absorption in a fluid. Various acoustic streaming patterns have been analysed in acoustofluidic manipulation devices due to different mechanisms of attenuation, most notably Eckart (1947) streaming and boundary-driven streaming (Nyborg 1958). The former is generated due to the energy dissipation in the bulk of a fluid, while the latter is formed from dissipation in an acoustic boundary layer (Wiklund et al. 2012). The streaming in both the cases is driven by spatial variation in the Reynolds stress (the mean value of the acoustic momentum flux). We refer to the driving term here as the Reynolds stress force (RSF) (Lighthill 1978).

**Fig. 1:**
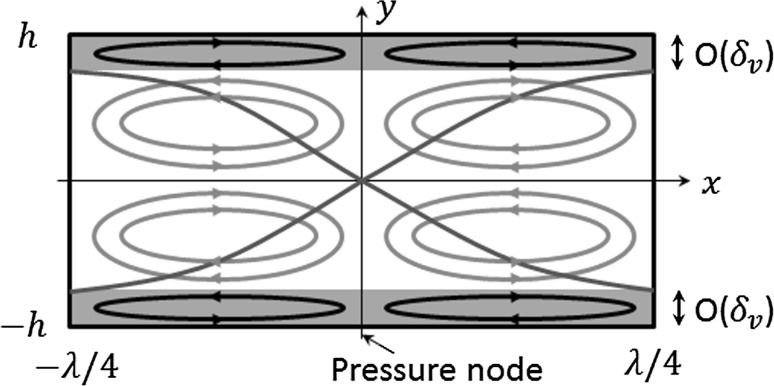
Schematic representation of the classical boundary-driven acoustic streaming flows in a two-dimensional rectangular channel, where $\lambda$ is the acoustic wavelength, $\delta_{v}$ is the thickness of the acoustic boundary layer and the *curves* represent the distribution of acoustic pressure magnitudes

Most bulk acoustic wave microfluidic manipulation devices utilise ultrasonic standing waves, where the scale of the fluid channel (in at least one dimension) is typically of the same order as the acoustic wavelength, meaning that the acoustic streaming fields are dominated by boundary-driven streaming as Eckart-type streaming generally needs longer distances to allow acoustic attenuation in the bulk of the fluid. Both types of streaming are typically found in surface acoustic wave (SAW) devices (Nama et al. 2015). The classical two-dimensional (2D) boundary-driven acoustic streaming fields are usually referred to as Rayleigh–Schlichting streaming (Rayleigh 1883; Schlichting 1932), recognising the contributions of Rayleigh and Schlichting to solving the acoustic streaming problems outside and inside the acoustic boundary layer region, respectively. Generally, their solutions describe the steady motion of periodic vortices within one-dimensional (1D) standing wave fields, comprising four pairs of counter-rotating vortices within each acoustic half-wavelength (Fig. 1). Subsequently, a series of modifications of Rayleigh’s solution have been proposed (Westervelt 1952; Nyborg 1953, 1958; Zarembo 1972; Riley 1998; Hamilton et al. 2003), many of which have been reviewed by Boluriaan and Morris (2003) and Valverde (2015). Moreover, many solutions for boundary-driven streaming patterns around obstacles have been proposed (Stuart 1965; Riley 1975, 1987, 1992; Amin and Riley 1990; Rednikov and Sadhal 2004, 2011) based on the streaming patterns observed around vibrating cylinders and spheres.

In most acoustofluidic particle manipulation devices, the acoustic streaming fields are considered as a disturbance as they place a practical lower limit on the particle size that can be manipulated by the primary acoustic radiation force (Barnkob et al. 2012; Bruus 2012a, b). However, acoustic streaming can also play an active role in such systems, such as particle trapping (Lutz et al. 2006; Chung and Cho 2008; Hammarstrom et al. 2012; Yazdi and Ardekani 2012; Hammarstrom et al. 2014), two-dimensional (2D) particle focusing (Antfolk et al. 2014) and particle separation (Devendran et al. 2014). Understanding the causal mechanisms of boundary-driven streaming flows is important in order to create designs for enhancing or minimising the streaming effects in acoustofluidic manipulation devices.

In recent decades, the rapid development of computational technology has allowed numerical simulations of acoustic streaming in models at the scale of practical experimental devices, making simulation an important tool in estimating the performance of acoustofluidic manipulation systems too complex for analytical solutions. On the one hand, 2D modelling of classical boundary-driven streaming has shown good consistency with theoretical solutions (Kawahashi and Arakawa 1996; Aktas and Farouk 2004) and with experimental measurements (Augustsson et al. 2011; Muller et al. 2012, 2013). On the other hand, most recently, computationally efficient methods have allowed three-dimensional (3D) simulations of boundary-driven streaming (Lei et al. 2013, 2014a, b, 2016; Hahn et al. 2015), enabling the demonstration of complex 3D characteristics of acoustic streaming flows and leading to an understanding of the driving mechanisms of streaming patterns experimentally observed in many practical acoustofluidic manipulation devices (Hagsater et al. 2007; Hammarstrom et al. 2012).

However, despite having shown good agreement between the modelling and experimental measurements and insight into previously puzzling phenomena, the scope of application of the numerical methods has not been fully established. In this paper, two numerical methods for the simulation of boundary-driven streaming in acoustofluidic devices are compared to analytical solutions for boundary-driven streaming in order to explore the conditions in which they can be applied to predict boundary-driven streaming fields to assist the design of acoustofluidic devices. By establishing this, boundary-driven streaming patterns in acoustofluidic devices can be solved using one or both of these two methods.

Section 2 presents the fundamental governing equations for acoustic streaming theory. In Sect. 3, two numerical methods for modelling boundary-driven streaming are explicitly described, including the equations solved, the boundary conditions required for each step of the numerical processes and the modelled results, including the driving mechanism of classical boundary-driven streaming and comparisons between these two numerical methods and Hamilton et al.’s (2003) analytical solution. Overall conclusions are drawn in Sect. 4.

## Basic theory of acoustic streaming

Before describing the two numerical methods, the fundamental governing equations of acoustic streaming theory are introduced. In the following, we use bold and normal emphasis fonts to represent vector and scalar quantities, respectively. Here, we assume a homogeneous isotropic fluid, in which the continuity and momentum equations for the fluid motion are:

1a $$\frac{\partial \rho }{\partial t} + \nabla \cdot \left( {\rho \varvec{u}} \right) = 0,$$

1b $$\rho \left( {\frac{{\partial \varvec{u}}}{\partial t} + \varvec{u} \cdot \nabla \varvec{u}} \right) = - \nabla p + \mu \nabla^{2} \varvec{u} + \left( {\mu_{b} + \frac{1}{3}\mu } \right)\nabla \nabla \cdot \varvec{u},$$

where $\rho$ is the fluid density, $t$ is time, $\varvec{u}$ is the fluid velocity, $p$ is the pressure, and $\mu$ and $\mu_{b}$ are, respectively, the dynamic and bulk viscosity coefficients of the fluid. It is well worth noting the meaning of each term in Eq. (1b). The left-hand side represents the inertia force per unit volume on the fluid, and the two terms in the bracket are the unsteady acceleration and convective acceleration of a fluid particle, respectively. The right-hand side indicates the divergence of stress, including the pressure gradient and the viscosity forces. Other forces, e.g. the gravity force, are not shown as they are generally negligible compared to the forces presented.

The two methods introduced in this paper are based on perturbation theory, which provides an excellent tool for bringing out the fundamental core of acoustic and streaming phenomena (Bruus 2012a, b; Sadhal 2012). It is assumed that the second-order acoustic streaming is superposed on the steady-state first-order acoustic velocity field. Following this theory, the fluid density, pressure, and velocity can, respectively, be expressed as:

2a $$\rho = \rho_{0} + \rho_{1} + \rho_{2} + \cdots ,$$

2b $$p = p_{0} + p_{1} + p_{2} + \cdots ,$$

2c $$\varvec{u} = \varvec{u}_{1} + \varvec{u}_{2} + \cdots ,$$

where the subscripts 0, 1 and 2 represent the static (absence of sound), first-order and second-order quantities, respectively.

Substituting Eq. (2) into Eq. (1) and considering the equations to the first order, Eq. (1) for solving the first-order acoustic velocity takes the form,

3a $$\frac{{\partial \rho_{1} }}{\partial t} + \rho_{0} \nabla \cdot \varvec{u}_{1} = 0,$$

3b $$\rho_{0} \frac{{\partial \varvec{u}_{1} }}{\partial t} = - \nabla p_{1} + \mu \nabla^{2} \varvec{u}_{1} + \left( {\mu_{b} + \frac{1}{3}\mu } \right)\nabla \nabla \cdot \varvec{u}_{1} .$$

Repeating the above procedure, considering the equations to the second order and taking the time average of Eq. (1) using Eq. (2), the continuity and momentum equations for solving the second-order time-averaged acoustic streaming velocity can be turned into

4a $$\nabla \cdot \overline{{\rho_{1} \varvec{u}_{1} }} + \rho_{0} \nabla \cdot \overline{{\varvec{u}_{2} }} = 0,$$

4b $$- \varvec{F} = - \nabla \overline{{p_{2} }} + \mu \nabla^{2} \overline{{\varvec{u}_{2} }} + \left( {\mu_{b} + \frac{1}{3}\mu } \right)\nabla \nabla \cdot \overline{{\varvec{u}_{2} }} ,$$

where the upper bar denotes a time-averaged value and $\varvec{F} = - \rho_{0} \overline{{\varvec{u}_{1} \nabla \cdot\varvec{u}_{1} + \varvec{u}_{1} \cdot\nabla \varvec{u}_{1} }}$ is the RSF (Lighthill 1978). The divergence-free velocity $\overline{{\varvec{u}_{2}^{\varvec{M}} }} = \overline{{\varvec{u}_{2} }} + \overline{{\rho_{1} \varvec{u}_{1} }} /\rho_{0}$, derived from Eq. (4a), is the mass transport velocity of the acoustic streaming, which is generally closer to the velocity of tracer particles in a streaming flow than $\overline{{\varvec{u}_{2} }}$ (Nyborg 1998).

Taking the curl of both sides of Eq. (4b), we establish that

5 $$\mu \nabla^{2} \left( {\nabla \times \overline{{\varvec{u}_{2} }} } \right) = - \nabla \times \varvec{F}.$$

It can be seen that the second-order streaming vortices are closely related to the rotationality of the RSFs. One advantage of this equation over Eq. (4b) is that the second-order pressure, $\overline{{p_{2} }}$, does not need to be considered. Thus, it can be established whether acoustic streaming vortices can be generated in a plane from the rotationality of the RSF field in that plane, although Nyborg (1965) points that for certain boundary conditions some knowledge of $\overline{{p_{2} }}$ is necessary to determine $\overline{{\varvec{u}_{2} }}$. The use of Eq. (5) to investigate the potential existence of streaming vortices is very useful to many problems, including the question of boundary-driven streaming in bulk acoustofluidic devices discussed here, in which the acoustic streaming fields usually appear as regular vortex patterns.

In the results shown later, this relationship will be used to elucidate the driving mechanism of classical boundary-driven streaming patterns and to draw out the key causal factors.

## Numerical methods, results and discussion

The numerical simulations were conducted in COMSOL 4.4 (2014). In this paper, we focus on Rayleigh–Schlichting streaming (as shown in Fig. 1) and the classical boundary-driven streaming in 2D water-filled rectangular channels, which is the most common type of acoustic streaming extensively discussed in the literature. Two methods are introduced and compared here in order to demonstrate their viability and applicability for the modelling of boundary-driven streaming fields in acoustofluidic systems of varying dimensions. For both methods, only the acoustic and streaming fields in the upper half of the rectangular channels were modelled as they are symmetric about $y = 0$ (Fig. 1).

**Fig. 2:**
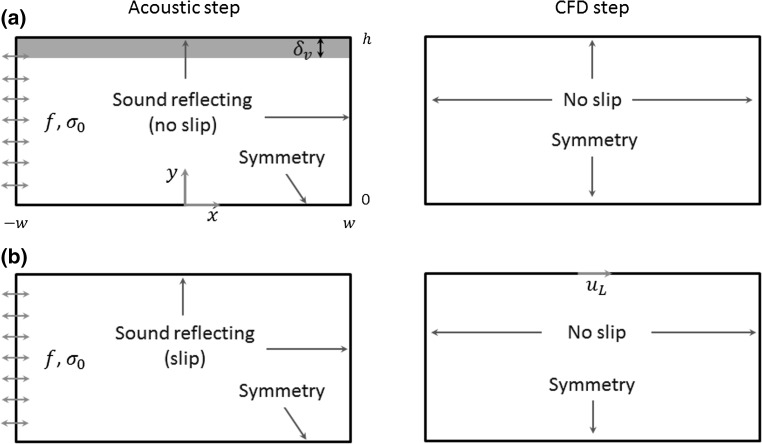
Comparisons of boundary conditions between the two numerical methods on the modelling of classical boundary-driven streaming fields in 2D rectangular channels: **a** the Reynolds stress method (RSM) and **b** the limiting velocity method (LVM), where $\sigma_{0}$ is the amplitude of harmonic oscillation, $f$ is its driving frequency, the *orange layer* is the acoustic boundary layer thickness ($\delta_{v}$, not to scale) and $u_{L}$ is the limiting velocity working as a slip velocity boundary condition on the edge where the acoustic boundary layer is ignored (colour figure online)

The required 1D standing wave fields in these rectangular channels were established by a harmonic excitation of the left boundaries at a frequency $f \approx 1$ MHz, as shown in Fig. 2. The thickness of the acoustic boundary layer in water at this frequency, $\delta_{v} = \sqrt {2\nu /\omega } \approx 0.6$ μm, where $\nu = \mu /\rho_{0}$ is the kinematic viscosity coefficient of the fluid and $\omega = 2\pi f$ is the angular frequency. Generally, it is found that the acoustic velocity decays from its maximum value to zero at the wall over a greater distance than $\delta_{v}$; in the results below and also in the work of Hamilton et al. (2003), this distance increases with the decrease in channel height, $h$. This will be discussed further below.

### The Reynolds stress method (RSM)

As illustrated, boundary-driven streaming originates from the dissipation of acoustic energy within the thin acoustic boundary layer region. The first method, the RSM, explores the origin of boundary-driven streaming flows, the net force on the fluid, and investigates how the boundary-driven streaming vortices inside and outside the acoustic boundary layer region are formed from the RSFs. The numerical process can be split into two steps:

1. Acoustic step: the first-order acoustic fields were solved, including the fine structure in the acoustic boundary layer. A COMSOL ‘*Thermoacoustics, Frequency Domain*’ interface was used to predict the first-order acoustic fields, which solves the acoustic pressure fields following (valid when $\left| {p_{1} } \right| \ll \rho_{0} c^{2}$ (Kinsler et al. 2000), where $\left| {p_{1} } \right|$ represents the acoustic pressure amplitude and $c$ is the sound speed):
  6 $$\nabla^{2} p_{1} + \frac{{\omega^{2} }}{{c^{2} }}p_{1} = 0,$$
  and the acoustic velocity fields using the relationships between the acoustic pressure and velocity fields derived from the inviscid form of Eq. (3).
  In this step, the left boundary of the rectangular channel was set as normal stress excitation, the bottom boundary was a symmetric condition and the remaining boundaries were sound reflecting conditions, as shown in Fig. 2a.
2. Computational fluid dynamics (CFD) step: the acoustic streaming fields were then solved. Here, a COMSOL ‘*Creeping Flow*’ interface was used to solve the second-order acoustic streaming fields following Eq. (4). The supplied ‘Creeping flow’ interface was edited to reflect Eq. (4), which initially does not include the first term in (a) and the last term in (b). In 2D Cartesian coordinates shown in Fig. 2a, the two components of the RSF $\varvec{F}$, ($F_{x} , F_{y}$), can be calculated from
  7a $$F_{x} = \rho_{0} \left( {\partial \overline{{u_{1}^{2} }} /\partial x + \partial \overline{{u_{1} v_{1} }} /\partial y} \right),$$
  7b $$F_{y} = \rho_{0} \left( {\partial \overline{{u_{1} v_{1} }} /\partial x + \partial \overline{{v_{1}^{2} }} /\partial y} \right),$$
  where $u_{1}$ and $v_{1}$ are the two components of the acoustic velocity vector $\varvec{u}_{1}$ along coordinates $x$ and $y$, respectively. In this step, all the edges were set as no-slip boundary conditions besides the symmetric condition of the bottom boundary, which are shown in Fig. 2b.

### The limiting velocity method (LVM)

It has been demonstrated previously that the time-averaged streaming velocity at the extremity of the inner streaming vortex (the limiting velocity) can be approximated as a function of the first-order linear acoustic velocity field outside the acoustic boundary layer as long as the radius of curvature of the surface is much greater than the acoustic boundary layer thickness (Nyborg 1958; Lee and Wang 1989). The streaming field in the bulk of the fluid (the outer streaming) is assumed to be driven by this limiting velocity field. Thus, the acoustic streaming fields outside the acoustic boundary layer region (the outer streaming) can be predicted, provided that the distribution of the first-order linear acoustic velocity field is known. This is referred to as the LVM here, and the numerical process for this method can also be split into two steps:

1. First, a COMSOL ‘*Pressure Acoustics, Frequency Domain*’ interface was used to model the first-order acoustic fields, which solves the harmonic, linearised acoustic problem, as described in Eq. (6). In this step, the left boundary of the chamber was considered as normal stress excitation, the bottom boundary was a symmetric condition and the remaining two edges were sound hard boundary conditions (this includes a slip velocity boundary condition, and thus, no boundary layers are created), which are described in Fig. 2c.
2. Then, a COMSOL ‘*Creeping Flow*’ interface was used to obtain the fluid motion, on which the limiting velocity, $u_{L}$, derived from the first-order acoustic velocity field, was applied as a slip velocity boundary condition on the top edge of the fluid channel, $y = h$. In the 2D models with their 1D standing wave field presented here, the limiting velocity equation can be approximated as
  8 $$u_{L} = \frac{3}{4\omega }u_{1}^{*} \frac{{{\text{d}}u_{1} }}{{{\text{d}}x}},$$
  where the superscript, *, represents the complex conjugate. Other boundary conditions are the same as for the RSM method. In this domain, the following equations were solved:
  9a $$\nabla \cdot \overline{{\varvec{u}_{2} }} = 0,$$
  9b $$\nabla \overline{{p_{2} }} = \mu \nabla^{2} \overline{{\varvec{u}_{2} }} .$$

As only outer streaming fields are solved in this method, with the assumption of low velocity, incompressible flow, the first term in the left-hand side of Eq. (4a) is zero, and thus, $\overline{{\varvec{u}_{2} }} = \overline{{\varvec{u}_{2}^{\varvec{M}} }}$ (Hamilton et al. 2003) (the last term in the right-hand side of Eq. (4b) is also zero) as we presented previously (Lei et al. 2013, 2014a, b, 2016). Then, as discussed by Lighthill (1978), the RSF in the bulk of the fluid can set up hydrostatic stresses, but in the absence of attenuation these will not create vortices; hence, these terms are not included in Eq. (9b). This is confirmed by noting that $\nabla \times \varvec{u}_{1} = 0$ in the model presented here which from Eq. (4) implies that the RSF is also irrotational.

### Mesh constitutions

In terms of channel dimensions, a series of channels with $h$ ranging from $\delta_{v}$ to 250 $\delta_{v}$ were considered, where the channel widths were the same, $2w = \lambda /2 = 0.74$ mm. All the model parameters are summarised in Table 1.

**Table 1:** Model parameters

Quantity	Abbreviation	Value	Unit
Density of water	$\rho_{0}$	998	kg m^−3^
Dynamic viscosity of water	$\mu$	0.893	mPa s
Bulk viscosity of water	$\mu_{b}$	2.47	mPa s
Speed of sound in water	$c$	1480	m s^−1^
Acoustic wavelength	$\lambda$	1.48	mm
Boundary layer thickness	$\delta_{v}$	~0.6	µm
Channel dimensions	$2w \times h$	$\lambda /2 \times \left( {\delta_{v} - 250\delta_{v} } \right)$	mm^2^

**Fig. 3:**
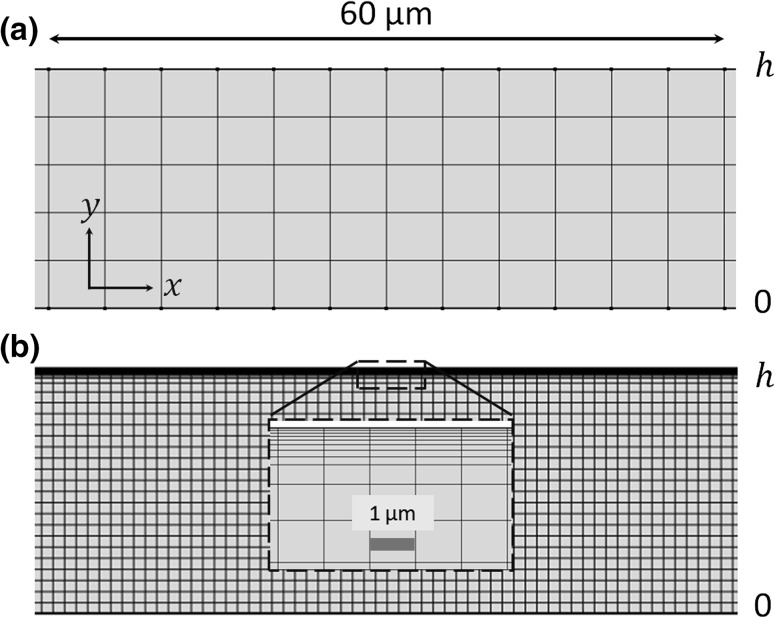
Examples of the mesh constitutions in rectangular channels for the two different methods: **a** the limiting velocity method and **b** the Reynolds stress method, where only a portion of the fluid channels in the $x$-direction (distance of 60 µm) is shown

**Fig. 4:**
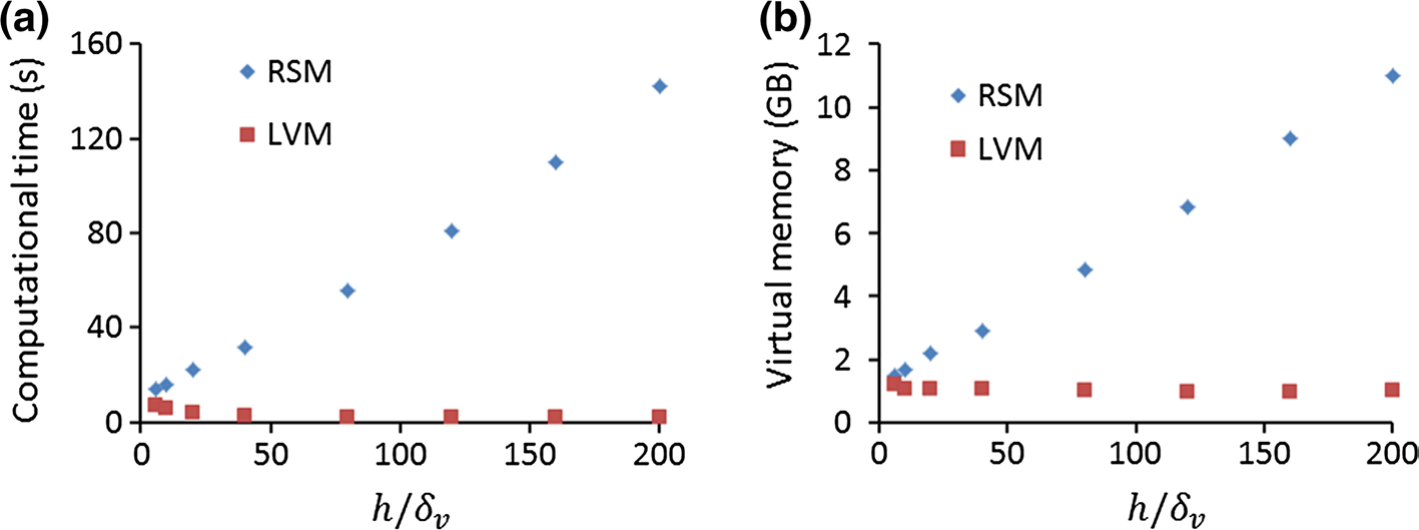
Comparisons of computational time (**a**) and virtual memory (**b**) required for the Reynolds stress method (RSM) and the limiting velocity method (LVM) in devices with various $h$. For $h/\delta_{v} = 200$, the LVM takes 2 s

As shown in Fig. 3, for the LVM, a uniform mesh along the channel height was used, while a boundary layer mesh near the top boundary of the fluid channel was used in order to resolve the acoustic and streaming fields in the acoustic boundary layer region for the RSM. Moreover, it can be seen that mesh sizes in the bulk of the fluid for the RSM should be small enough to keep the mesh size continuity inside and outside the acoustic boundary layer. A mesh size-dependency study was firstly conducted in order to determine the mesh sizes required for each case for high accuracy. It was found that as few as two elements in the $y$-direction are sufficient to accurately capture the streaming pattern and magnitude using the LVM. However, in order to allow for cases that are more complex than the simple one explored in the dependency study we used a higher density of five elements in the $y$-direction, which makes it suitable for both 2D and 3D modelling of outer streaming flows. For the RSM, however, smaller mesh elements (first layer of $\sim\delta_{v} /5$) are required to resolve the acoustic and streaming fields inside the acoustic boundary layer, and tens of layers of mesh elements in the $y$-direction are needed in order to obtain smooth distributions of acoustic and streaming fields across the channel heights ($y$-direction). Based on the mesh constitutions described above, the computational time and virtual memory required for the RSM and the LVM in devices with various channel heights are compared in Fig. 4 (performed on a Lenovo Y50 running Windows 8 (64-bit) equipped with 16 GB RAM and Intel(R) Core(TM) i7-4710HQ processor of clock frequency 2.5 GHz). It can be seen that the RSM is a far more computationally expensive method and is less suitable for 3D modelling of large-scale devices, where the length scale of the fluid channels is orders of magnitude larger than the acoustic boundary layer thickness (the case for most practical experimental acoustofluidic manipulation devices).

### First-order acoustic fields

**Fig. 5:**
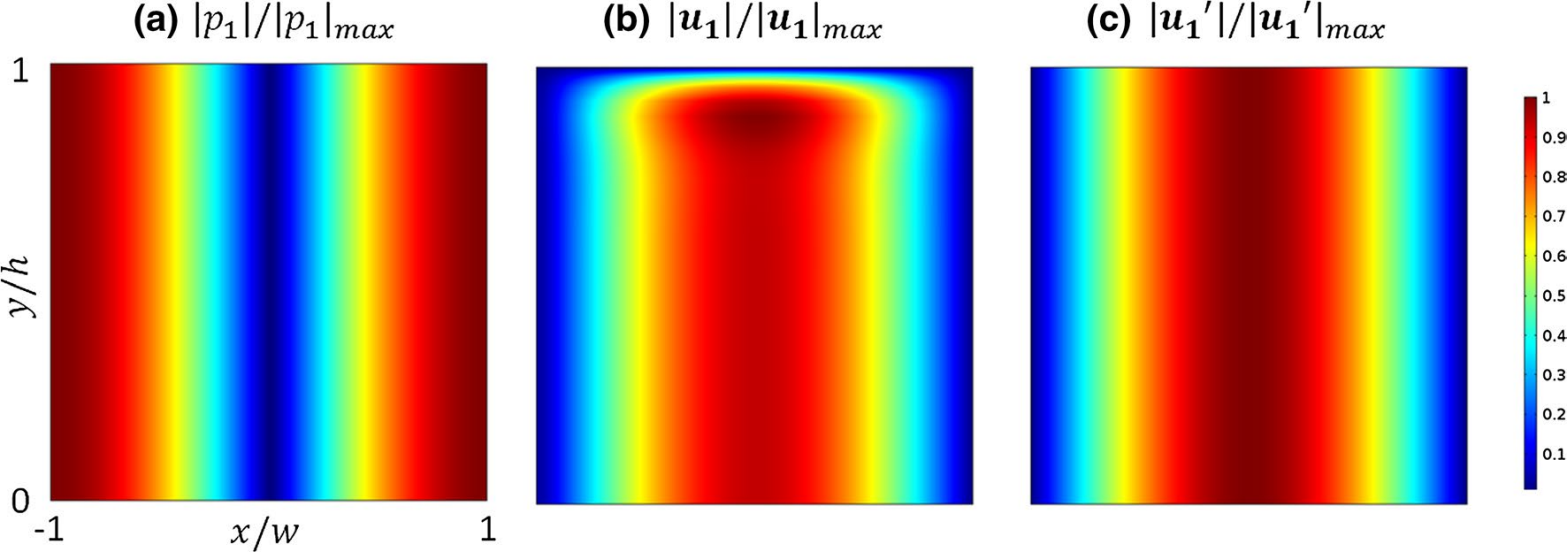
Modelled first-order acoustic pressure and velocity fields: **a** distribution of the normalised acoustic pressure magnitude (both RSM and LVM methods); **b** RSM: distribution of the normalised acoustic velocity magnitude ($h = 40\delta_{v}$) solved from the COMSOL ‘*Thermoacoustics, Frequency Domain*’ interface; **c** LVM: distribution of the normalised acoustic velocity magnitude solved from the COMSOL ‘*Pressure Acoustics, Frequency Domain*’ interface, where $\left| \cdot \right|_{\hbox{max} }$ represents the maximum amplitude in the cavity

The modelled first-order acoustic fields are shown in Fig. 5. It can be seen that, for both methods, a 1D half-wavelength standing wave field was established in the $x$-direction of the chambers (Fig. 5a) with a pressure node at the centre ($x = 0$) and antinodes at the two ends ($x = \pm w$) (the model was run at the resonant frequency, which was chosen to obtain the maximum energy density in the channel). However, the modelled acoustic velocity fields vary between these two models. Figure 5c plots the acoustic velocity field modelled from the LVM. It can be seen that the velocity and pressure fields are uniform along the $y$-axis and have a 90° phase difference. The modelled acoustic velocity field from the RSM is shown in Fig. 5b, where an acoustic boundary layer near the top boundary, $y = h$, is seen. It can be seen that the acoustic velocity magnitude increases rapidly from 0 at the top boundary, $y = h$, to its maximum value and then decreases a little in magnitude to the constant value found in the bulk of the channel.

### Second-order acoustic streaming patterns

**Fig. 6:**
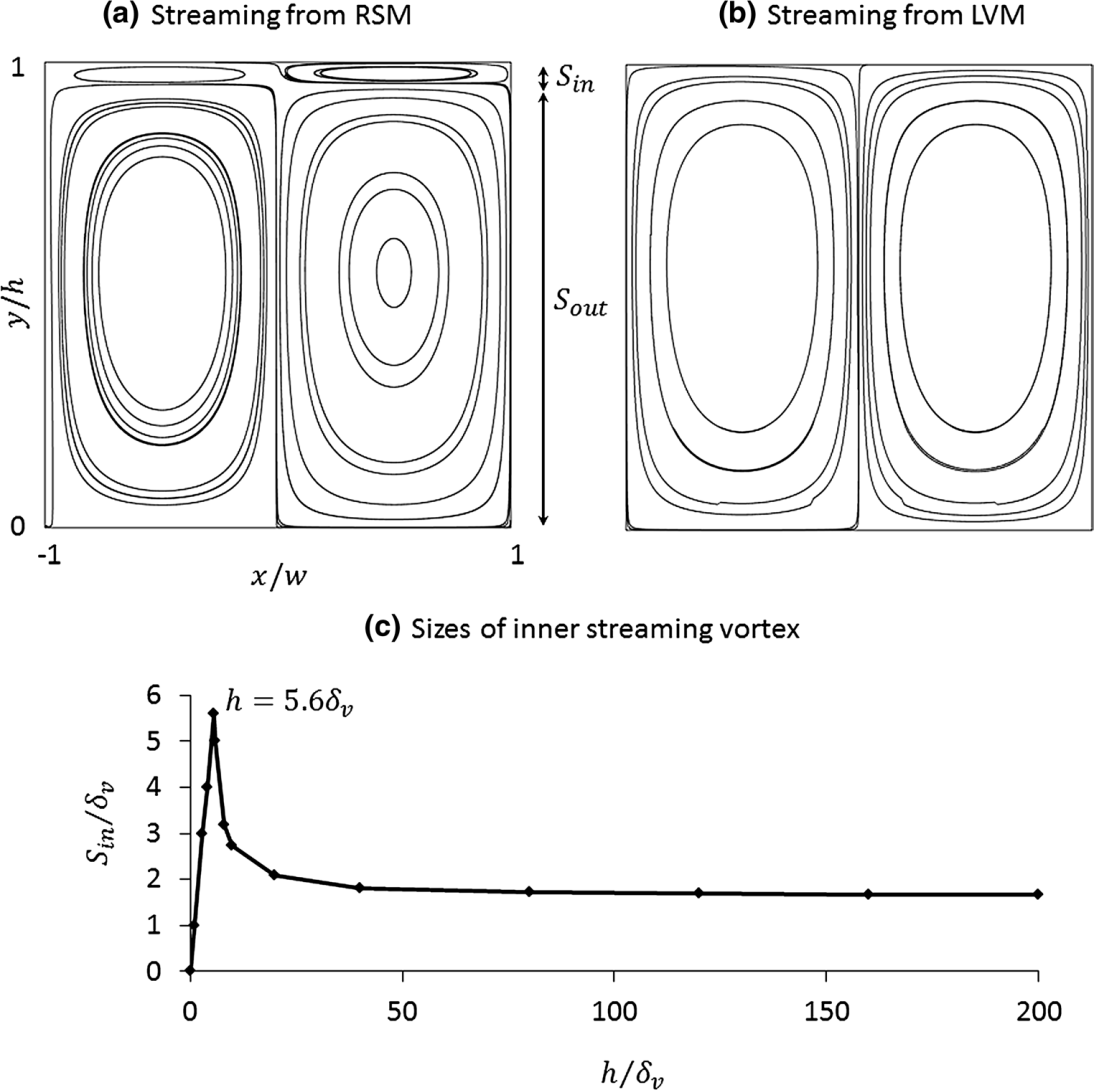
Modelled boundary-driven streaming patterns: **a** from the Reynolds stress method (RSM); **b** from the limiting velocity method (LVM); and **c** the $y$-extent of the inner streaming vortex in devices with various $h$, where $\delta_{v}$ is the thickness of acoustic boundary layer and $S_{\text{in}}$ and $S_{\text{out}}$ represent the sizes of inner and outer streaming vortices, respectively

The modelled acoustic streaming patterns are shown in Fig. 6. Figure 6a, b plots the acoustic streaming patterns modelled from these two methods in a channel where $h = 40\delta_{v}$. In the RSM method, the $y$-extent of the inner and outer acoustic streaming vortices are labelled, respectively, $S_{\text{in}}$ (defined here as the distance from the boundary to the location at which streamlines switch between clockwise and anticlockwise directions) and $S_{\text{out}}$ as shown in Fig. 6a. The sizes of the inner streaming vortex, $S_{\text{in}}$, in devices with various channel heights are plotted in Fig. 6c, which shows that $S_{\text{in}}$ scales linearly with the growth of $h$ in devices where $h \le 5.6\delta_{v}$ as only inner vortices were obtained in the entire chamber, which is close to the value found from Hamilton et al.’s analytical solution (Hamilton et al. 2003), which is about $5.7\delta_{v}$. With further increases of $h$ an outer vortex appears, and $S_{\text{in}}$ drops and soon stabilises to a value of $\sim\delta_{v}$, as the condition $h \gg \delta_{v}$ is obtained; this is shown in Fig. 6c. Investigating the cause of this change, which is explored in more detail below, we find that in the cases where there are both inner and outer streaming vortices, the $y$-extent of the inner vortex closely follows the distance from the wall to the position of maximum acoustic velocity. However, due to the approximations of the LVM, this behaviour at small $h$ is not seen, and this method always gives the same streaming patterns in channels of different channel heights, leading to substantial errors for small $h.$

### Comparisons on the streaming velocities

**Fig. 7:**
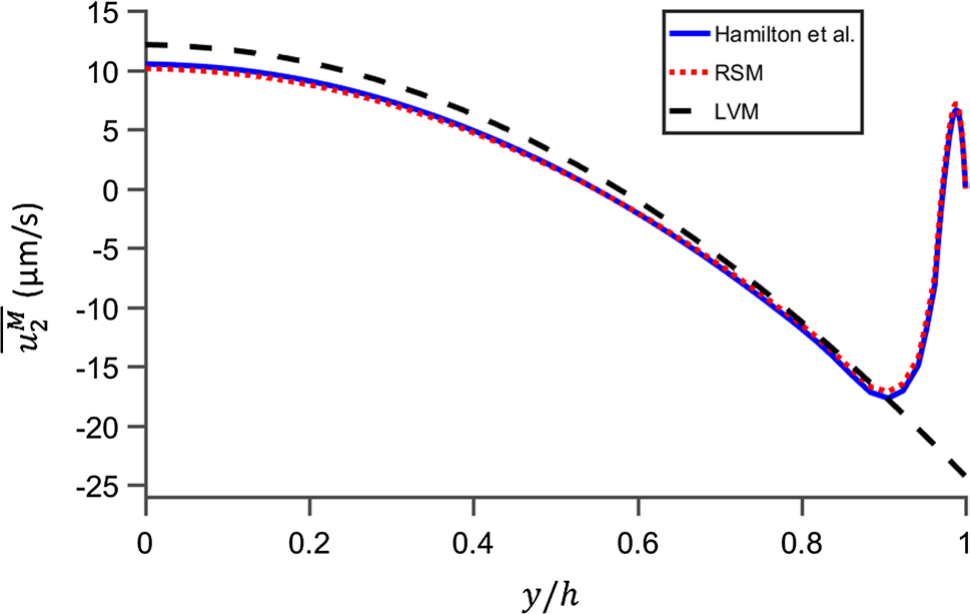
Comparisons of the vertical distributions of the modelled $x$-component acoustic streaming velocities, $\overline{{u_{2}^{M} }}$, along line $x = - w/2$ between the Reynolds stress method (RSM), the limiting velocity method (LVM) and Hamilton et al.’s solution (Hamilton et al. 2003) in a chamber with $h = 40\delta_{v}$. The streaming velocity magnitudes were obtained from an acoustic pressure amplitude of 0.46 MPa

In order to demonstrate the accuracy of these two methods, the modelled acoustic streaming velocity magnitudes were compared to Hamilton et al.’s analytical solution (Hamilton et al. 2003) in 2D rectangular chambers, as shown in Fig. 7, where the vertical distributions of the $x$-component streaming velocity, $u_{2}$, along $x = - w/2$ are plotted. In this graph, the excitation amplitude has been adjusted to give a first-order acoustic pressure field amplitude of 0.46 MPa in order to compare the acoustic streaming velocity fields. It can be seen that the distribution and magnitudes of streaming velocities modelled from the RSM are in good accordance with those obtained from Hamilton et al.’s solution (Hamilton et al. 2003).

**Fig. 8:**
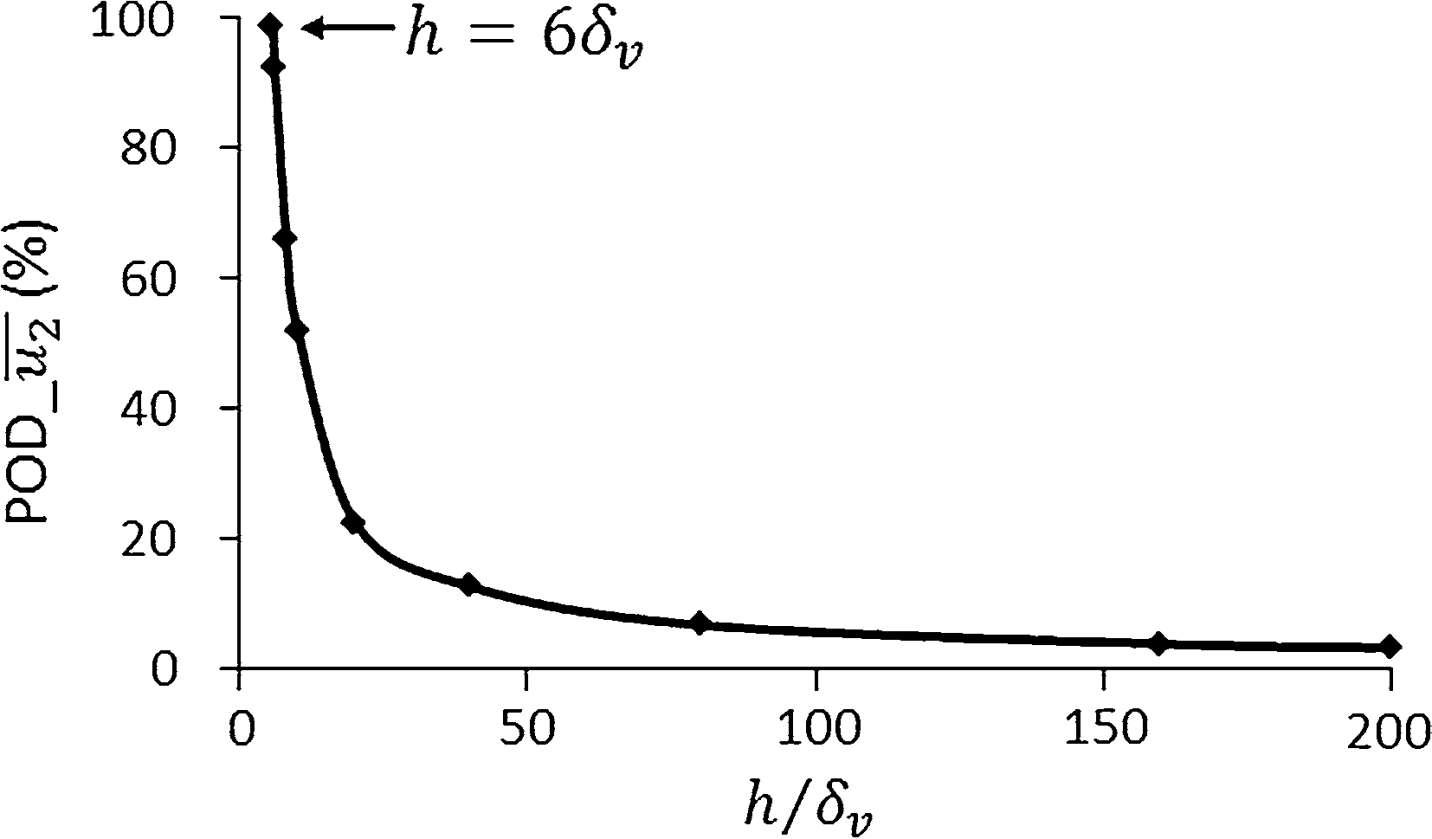
Comparisons of the modelled acoustic streaming velocities in the bulk of the fluid by two methods, the Reynolds stress method (RSM) and the limiting velocity method (LVM). POD_ $\overline{{u_{2} }}$ represents percentage of difference on the $x$-component acoustic streaming velocity, $\overline{{u_{2} }}$, at point ($- w/2$, 0). POD_ $\overline{{u_{2} }} = \left( {\overline{{u_{2l} }} - \overline{{u_{2r} }} } \right)/\overline{{u_{2l} }}$, where $\overline{{u_{2l} }}$ and $\overline{{u_{2r} }}$ are the streaming velocities obtained from the LVM and the RSM, respectively. $\delta_{v}$ is the thickness of the acoustic boundary layer

Turning to examine the outer streaming vortex, we first note that in devices where $h \le 5.6\delta_{v}$ only inner streaming vortices exist in the whole chamber, and under such circumstances, the LVM is not suitable. For the case presented in Fig. 7 ($h = 40\delta_{v}$), the modelled magnitude of streaming velocity was found to be approximately 14% higher from the LVM than that from the RSM at the centre of the fluid channel ($y = 0$). The errors for channel heights ranging from 6 $\delta_{v}$ to 200 $\delta_{v}$ are shown in Fig. 8. For each case, the percentage of difference between these two methods, on the modelled $x$-component streaming velocity magnitude, at point ($- w/2$, 0) was calculated:

10 $${\text{POD}}\_\overline{{u_{2} }} = \left( {\overline{{u_{2l} }} - \overline{{u_{2r} }} } \right)/\overline{{u_{2l} }} ,$$

where $\overline{{u_{2l} }}$ and $\overline{{u_{2r} }}$ are the time-averaged streaming velocities obtained from the LVM and the RSM, respectively.

Figure 8 shows that the difference in the streaming velocity magnitudes modelled by these two methods tends to be smaller with the increase in channel heights. We thus do not recommend applying the LVM to model the boundary-driven streaming in devices where the channel heights are below 100 $\delta_{v}$, which introduces around 5% of error on the magnitudes of acoustic streaming velocities. However, in most practical experimental acoustofluidic manipulation devices (not including SAW devices, see below), where 1D or 2D standing wave fields are established and 3D models are required to solve the acoustic and streaming fields, the LVM can be effectively applied as the channel dimensions are usually many orders of magnitude larger than the acoustic boundary layer thickness and typically only the acoustic streaming fields outside the acoustic boundary layer are of interest. This can further explain the good consistency between the experimental measurements in acoustofluidic manipulation devices and the results simulated from the LVM in 3D models presented in the literature recently (Lei et al. 2013, 2014a, b, 2016; Hahn et al. 2015).

### Driving mechanism of classical boundary-driven streaming

In the previous section, it was shown that in devices where $h \gg \delta_{v}$, the size of the inner vortex remains stable at approximately $1.7\delta_{v}$ (Fig. 6). This raises the question of why the inner streaming vortex is limited to the boundary layer region and does not have a container scale (i.e. size of $h$); in other words, what the cause of the vortex pairs inside and outside the boundary layer region is. While the answer to this is implicit in the well-established analytical solutions to the problem, it is instructive to examine the fields in more detail to see which components of the solution dominate the behaviour that is seen and hence approach a more causal explanation. In this section, we address this by investigating how the RSF fields vary inside and outside the boundary layer region.

**Fig. 9:**
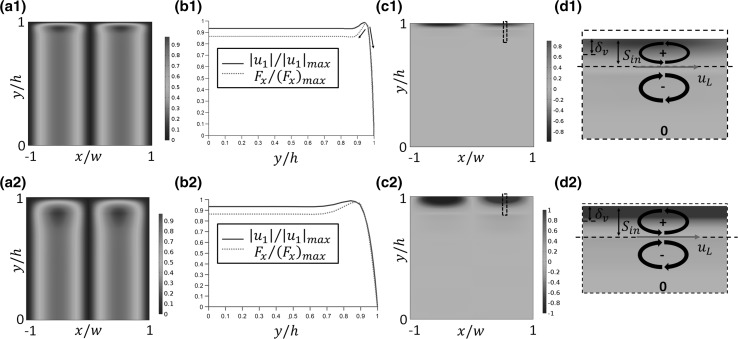
Modelled results in devices where $h = 40\delta_{v}$ (*first row*) and $h = 15\delta_{v}$ (*second row*): **a** the distribution of normalised Reynolds stress force magnitude, $\varvec{F}$; **b** the vertical distribution of normalised $\left| {u_{1} } \right|$ (*solid line*) and $F_{x}$ (*dotted line*) along $x = w/2$, where $\left| \cdot \right|_{\hbox{max} }$ represents the maximum absolute value; **c** the distribution of normalised $\nabla \times \varvec{F}$; and **d** the distribution of $\nabla \times \varvec{F}$ near the top boundary in the *dashed box* shown in (**c**), where $\delta_{v}$ is the thickness of acoustic boundary layer, $S_{\text{in}}$ shows the size of inner streaming vortex, $u_{L}$ is the limiting velocity that drives the outer streaming vortex, 0, −, and + represent the magnitudes of $\nabla \times \varvec{F}$ and the curved arrows represent the streaming pattern driven by the rotationality of $\varvec{F}$ in the $xy$ plane

Figure 9a1–d1 shows the RSF field in a channel where $h = 40\delta_{v}$, where all the quantities have been normalised by their maximum magnitudes to highlight their distributions. Here, we define another parameter $y^{\prime}$ as

11 $$y^{\prime} = h - y,$$

to describe the vertical distance to the top boundary ($y = h$).

Figure 9a1 shows that away from the upper boundary the RSF field has a sinusoidal distribution along the standing wave ($x$-direction); this will give rise to a hydrostatic pressure field in the bulk of the fluid (Lighthill 2001). Examining a slice of the field in the vertical direction ($y$), as shown in Fig. 9b1, the force field has a similar distribution to the $x$-component of the acoustic velocity. Consistent with the acoustic boundary layer, the magnitude of this velocity rises rapidly as $y^{\prime}$ increases (that is moving away from the boundary at $y = h$), reaches a peak and then decreases a little to a constant value in the bulk of chamber. Here, only $F_{x}$ is shown as the $y$-component force, $F_{y}$, is typically thousands of times smaller due to the 1D standing wave only creating an acoustic boundary layer in the $y$-direction being established in the $x$-direction of the chamber (modelled results do not show significant variation on removing this component, supporting our emphasis on $F_{x}$). Moreover, under this approximation, the curl of the RSF can be approximated to

12 $$\nabla \times \varvec{F} \approx - \frac{{\partial F_{x} }}{\partial y}\varvec{k},$$

where $\varvec{k}$ is the unit vector perpendicular to the $xy$ plane. This means that, in devices in which a 1D standing wave is established along the $x$-axis of the chamber, the $y$-derivative of $F_{x}$ determines the rotationality of the RSF and hence streaming in the $xy$ plane. Considering the distribution of $F_{x}$ along the channel height (as shown in Fig. 9b1), two main force gradients can be found in the acoustic boundary layer region, shown with arrows, and it is these which determine the rotationality of $\varvec{F}$ in the near-boundary region and the boundary between the inner and outer streaming vortices.

The distribution of $\nabla \times \varvec{F}$ in the whole chamber is shown in Fig. 9c1, and a magnification of $\nabla \times \varvec{F}$ near the top boundary in the dashed box is presented in Fig. 9d1, where ‘+’ and ‘−’ signs were used to show its direction. In the bulk of the fluid chamber, the RSF is nearly irrotational. The force field is rotational in regions close to the acoustic boundary layer with different directions in areas inside the boundary layer and that immediately outside it. Thus, a pair of oppositely rotating vortices is generated with a boundary close to the acoustic boundary layer. The streaming velocity at the juncture of these two vortices is the limiting velocity, shown as $u_{L}$ in Fig. 9d1. It is also the different rotationality on the RSF inside the boundary layer and that immediately outside it that determines the $y$-extent of inner streaming vortices ($S_{\text{in}}$), localising it in the thin boundary layer with a size of $O\left( {\delta_{v} } \right)$. In Fig. 9a2–d2, a second case is shown where the reduced chamber height leads to an increased distance from the boundary to the maximum acoustic velocity and hence thicker inner streaming vortex. Some model parameters and results shown in Fig. 9 are summarised in Table 2.

**Table 2:** Model parameters and results shown in Fig. 9, where $\delta_{v}$ is the acoustic boundary layer thickness, $f_{r}$ is the resonant frequency and $y^{\prime}$ is the vertical distance to the top boundary

$h$	$y^{\prime}$ for $\left| {u_{1} } \right|_{\hbox{max}}$	$y^{\prime}$ for $\left( {F_{x} } \right)_{\hbox{max}}$	$S_\text{in}$
40 $\delta_{v}$	1.94 $\delta_{v}$	1.94 $\delta_{v}$	1.94 $\delta_{v}$
15 $\delta_{v}$	2.31 $\delta_{v}$	2.08 $\delta_{v}$	2.31 $\delta_{v}$

## Conclusion

Two numerical methods for the modelling of boundary-driven streaming fields in acoustofluidic manipulation devices have been compared in this paper, to provide guidance on choosing appropriate methods to predict acoustic streaming patterns in experimental devices and to assist in selecting device designs to optimise their performances.

It was shown that the RSM can accurately model the inner and outer streaming patterns and the magnitudes of streaming velocities in good accord with analytical solutions. The generation mechanism of classical boundary-driven streaming in 2D rectangular chambers was elucidated by examining its driving forces, the RSF. It is the different rotationality of the RSF inside and immediately outside the boundary layer that forms the inner and outer streaming vortices and forces the former in the thin acoustic boundary layer with a size of approximately $\delta_{v}$ in devices where $h \gg \delta_{v}$. This understanding of the mechanism could also be extended to better understand (and hence design) boundary-driven streaming flows in diverse types of acoustofluidic channels, including those with non-flat fluid channel surfaces (Lei et al. 2014a, b; Nama et al. 2014; Ovchinnikov et al. 2014). However, although the RSM has shown high precision for modelling both acoustic streaming patterns and magnitudes of streaming velocities, the tiny mesh element required to resolve the acoustic and streaming fields in the near-boundary region suggests it may be a very computationally demanding method and thus not suitable for 3D modelling of most practical acoustofluidic manipulating devices.

The other method, the LVM, is more computationally efficient. The error it introduces to the magnitudes of streaming velocities rises with the fall of the channel dimensions such that it is not recommended for predicting the outer acoustic streaming velocities in devices where $h < 100\delta_{v}$. However, The LVM can be effectively applied to solve both 2D and 3D boundary-driven streaming fields in most practical experimental acoustofluidic manipulation devices where the channel dimensions are generally several orders of magnitude larger than the acoustic boundary layer thickness. For the LVM to be applicable, the surfaces of the fluid chambers must also satisfy the condition that the radius of curvature of the channel surfaces should be much greater than the thickness of the acoustic boundary layer.

It is noteworthy that these methods can also be applied to predict boundary-driven streaming fields in SAW devices and to analyse its contribution to the overall acoustic streaming fields in such devices. However, in such cases (and other situations where boundary motion is significant), care must be taken to accurately model the moving surface boundary condition, which is non-trivial in an Eulerian formulation (Koster 2007; Vanneste and Buhler 2011; Nama et al. 2016). In such devices where the length scales of fluid channel cross sections are typically orders of magnitude larger than the acoustic wavelength, Eckart-type streaming is likely to dominate the overall streaming fields (Ding et al. 2013; Yeo and Friend 2014). However, in other cases where the length scales of the fluid channel cross sections are of the same order of acoustic wavelength (e.g. Guo et al. 2015; Nama et al. 2015), boundary-driven streaming generated from the energy attenuation in the acoustic boundary layer region due to non-slip boundaries may be comparable or have a larger contribution to the overall streaming field.
